# Superb feeding behavior of *Aedes albopictus* transmitting Zika virus

**DOI:** 10.1371/journal.pone.0184871

**Published:** 2017-09-28

**Authors:** Young Ran Ha, Jun Ho Kim, Jeongeun Ryu, Sang Joon Lee

**Affiliations:** 1 Center for Biofluid and Biomimic Research, Pohang University of Science and Technology, Pohang, Republic of Korea; 2 Department of Mechanical Engineering, Center for Biofluid and Biomimic Research, Pohang University of Science and Technology, Pohang, Republic of Korea; Universite Francois-Rabelais de Tours, FRANCE

## Abstract

Disease-mediated mosquitoes have been receiving much attention, as the World Health Organization recently declared the Zika virus a global public health emergency. Mosquitoes transmit pathogens that cause various tropical diseases including malaria, dengue fever and yellow fever as well as Zika virus. The vector efficiency of mosquitoes depends on their blood-feeding characteristics and the mechanics of their blood-sucking pump system, but only a few studies have attempted to investigate these key issues. In this study, we demonstrate the rapid and gluttonous liquid-feeding characteristics of *Ae*. *albopictus* which transmits Zika virus can be explained by similar proportion of two blood-sucking pumps and accelerated liquid intake driven by fast expanding of pumps. Our results provide insight into the vector efficiency of *Ae*. *albopictus* in terms of feeding velocity, pumping frequency, liquid-intake rate, and wall shear stress.

## Introduction

Mosquitoes transmit various pathogens that cause infectious tropical diseases including dengue fever, malaria, filariasis, and Zika virus [1], which was declared a public health emergency of international concern on Feb 2016 [2]. The World Health Organization estimates that there are currently 500,000 to 1.5 million cases of Zika in the Americas and that it is spreading explosively [3].

The *Aedes albopictus* mosquitoes, which are found in the USA, southern Europe, Asia, and South Korea, also transmit Zika virus [2,4]. This species has been known as a very aggressive biter [5]. It also transmits Japanese encephalitis, West Nile virus, yellow fever, and dengue virus [5–6]. *Aedes togoi* occurs from subarctic to subtropic area along the coasts of the East Asian contries [7]. It is mainly involved in the transmission of numerous species of filariae, yellow fever, and Japanese encephalitis [8–10].

The vector efficiency of mosquitoes could be related to their blood-feeding behaviors [10–11]. A previous study investigated nectar-feeding of *Ae*. *albopictus* and *Ae*. *togoi* mosquitoes [12]. This nectar-feeding was appeared to be gradually activated within days after emergence. The feeding rate of *Ae*. *togoi* reached a peak value of 97.9% on day 9 after emergence. On the other hand, both male and female *Ae*. *albopictus* reached 80–90% feeding rate while they had been fed during 8hr after the nectar-feeding [12]. However, the mechanisms underlying these liquid-feeding behaviors remain unclear.

The blood-feeding behaviors are known to be controlled by two pump organs in the head, the cibarial dilator pump (CP) and the pharyngeal dilator pump (PP) [13]. The systaltic motions of the CP and PP generate high differential pressure for sucking highly viscous blood [14]. Therefore, the actions of these pumps combined with salivation directly affect the efficiency of pathogen delivery by mosquitoes [10,15–16]. During the blood-feeding process, pathogen intrusion can be influenced by probing behavior, blood intake rate, feeding persistence, and locomotor activity [10–11,17–19]. In addition, wall shear stress (WSS) is an important pathogenic factor that affects the transport of blood through conduits and all substances through vessel walls located near the tip of the food canal [10]. The adhesion and movement of the pathogens are critical for reproduction [20]. In our previous study, *Ae*. *togoi* generates higher feeding pressure with bigger volume variation of pumping organs, compared with *Ae*. *sinensis* [10]. In addition, *Ae*. *togoi* has high WSS in the feeding process. The high WSS is beneficial for removing pathogen attached to the host’s vessel wall [21–22]. However, the relationship between the high WSS of *Ae*. togoi and the mosquito’s vectorial capacity has not been well known yet. Unlike *Ae*. *togoi*, mechanical characteristics of *Ae*. *albopictus*, which are closely related to the feeding behaviors and pathogen transmission, remain poorly understood.

In this study, the feeding pump organs and liquid-feeding mechanics of *Ae*. *albopictus* were experimentally investigated and compared with those of *Ae*. *togoi*. The 3D morphological structures of the pumping organs of *Ae*. *albopictus* and *Ae*. *togoi* were examined using synchrotron X-ray microscopic computed tomography (SR-μCT). The volumetric variations and proportions of the blood-feeding pumps of the two *Aedes* mosquitoes were quantitatively compared. Blood-feeding parameters, such as flow rate (Q), WSS, and pumping frequency were measured in the two *Aedes* mosquitoes using a micro-particle image velocimetry (micro-PIV) technique to distinguish their vector efficiencies with respect to the blood-feeding flow characteristics. In addition, 2D X-ray images of the systaltic movements of the two pump organs were also investigated to examine their phasic volumetric variations and reveal the feeding mechanism of *Ae*. *albopictus* mosquitoes. These experimental results will elucidate the blood-feeding characteristics of *Ae*. *albopictus* in comparison with those of *Ae*. *togoi*.

## Methods

### Mosquito rearing and sample preparation

Following established rearing procedures [23], mosquitoes (*Aedes albopictus* (Diptera: Culicidae) and *Aedes togoi* Theobald 1907) were fed a 10% sugar solution at 27°C, 80% humidity and a 16 h: 8 h light/dark cycle. Mosquitoes were provided by Korea Centers for Disease Control and Prevention. Larvae were fed a slurry of ground fish food and baker’s yeast once a day. Approximately 150 to 200 larvae were reared in a 2L plastic container before the beginning of pupation. After pupation, the mosquitoes were transferred to a cage and provided a 10% sucrose-soaked cotton rod. The time of pupation was approximately 2 days.

### Synchrotron X-ray microscopic computed tomography (SR-μCT)

All SR-μCT experiments were conducted at the Biomedical Imaging beamline of Pohang Light Source-II in Korea. A white beam emitted from the multi-pole wiggler source of the 3-GeV storage ring was filtered through 1-mm-thick graphite after passing through a double multilayer monochromator that provided photon energies in the range of 10 KeV to 50 KeV. The optimal monochromatic X-ray energy for the tomographic scanning experiments in this study was experimentally determined to be approximately 24 KeV. The detector was positioned 20 cm downstream from the test sample to capture phase-contrast images. The monochromatic X-ray images transmitted through the test sample were consecutively recorded using an imaging detector (Andor Zyla, Andor, Oxford Instruments, Belfast, UK) with a spatial resolution of 2560 × 2160 pixels, upon which a YAG:Ce (30-μm thick) scintillation crystal was adhered. The captured X-ray images were converted on the scintillator into visible images. Each mosquito sample was immersed in ethanol and placed at the tip of a heat-sealed pipette made of polypropylene, which has a comparatively low X-ray absorption coefficient [24]. Parafilm was used to prevent the alcohol from evaporating. Each test sample was placed on a rotating stage, and 2D tomographic slice images of the samples were captured by rotating the stage at 0.5°-intervals from 0° to 180°. The 3D morphological structure of each mosquito sample was reconstructed from the captured tomographic slice images using Octopus image-processing software. To obtain a 3D volumetric image of the sample, a filtered back-projection algorithm was applied to the images. Cross-sectional images were stacked to animate a 3D structural image. The stacked images were rendered to show the 3D morphological structure using Amira® 5.3.3 image-analysis software (Visualization Sciences Group, Burlington, MA, USA).

### Scanning electron microscopy

Scanning electron microscopy (SEM) was employed to illustrate the morphological configurations of the mosquito samples tested in this study. Mosquito specimens were prepared by air-drying. The samples were then Ag-coated using a coater (Quorum Technology, SC7640 mode, East Sussex, United Kingdom) and examined by a field emission SEM (XL30S FEG, Philips Electron Optics B.V., the Netherlands) connected to an EDXS system at an acceleration voltage of 5 kV.

### Velocity and flow measurements

To quantitatively visualize the flow in the food canal of the two mosquito species, a micro-PIV technique was used. More than 50 female mosquitoes of similar size were used to acquire experimental information for each mosquito species. To visualize feeding liquid-flow in the food canal of a female mosquito, the opaque cuticle was removed by microsurgery.

A 1% (w/w) sucrose solution (density; ρ = 1002.1kg/*m*^3^, dynamic viscosity; *μ* = 0.927 × 10^−6^*m*^2^/*s*) at 25°C was used as the working flow. Kikuchi and Mochizuki measured blood flow rate (Q) in the food canal of a female mosquito and compared with their previous result acquired by using a sucrose solution. The velocity profiles in the food canal are almost parabolic, regardless of feeding liquid [25]. This implies that the blood-feeding flow in a female mosquito’s food canal can be considered as a Hagen-Poiseuille flow.

To obtain fluorescent images of the feeding flow, 1.0 μm-diameter fluorescent particles (Molecular Probes, Eugene, OR, USA) were diluted with the working fluid. An Nd:YAG laser (λ = 532 nm, SLOC, Shanghai. China) was used as a light source for the PIV system to extract information related to the blood-sucking velocity field with high accuracy. The fluorescent image (λ = 554 nm) of the tracer particles was passed through an objective lens (M = 20, N.A. = 0.5), and after high-pass filtering with an optical filter (λ = 550 nm), it was attached to the front of the microscope (Eclipse 80i, Nikon, Tokyo, Japan). Flow images were consecutively recorded by a high-speed camera (Fastcam SA1.1, Photron, USA). We captured consecutive images at a frame rate of 10,000frame*s*^−1^. The field of view is 256μm × 128μm and the spatial resolution of the PIV system is about 1μm/pixel. The interrogation window size is 16 × 8pixels. Personal computer was used to store the captured flow images and process the data. To estimate whether the tracer particles reliably represented the flow, the Stokes number, St, (*ρ*_*p*_*a*^2^*U*_*mean*_/18*μD*_*h*_) was calculated, where *ρ*_*p*_ is the density of the tracer particles; *a*^2^ is the diameter of the tracer particle; *U*_*mean*_ is the mean velocity of the flow; *μ* is the dynamic viscosity of the working fluid; and *D*_*h*_ is the internal diameter of the food canal. The Stokes number was estimated to be approximately 10^−3^, which is much smaller than 1. This estimate indicates that the tracer particles can be used as reliable flow tracers. All experiments were conducted under the same environmental conditions (25°C, 45% RH).

### Statistical analysis

All data were statistically analyzed using t-tests with 95% confidence intervals in SPSS (IBM, Chicago, IL, USA).

## Results

### Morphological differences of the proboscises and heads of two different female mosquitoes

**Fig 1:**
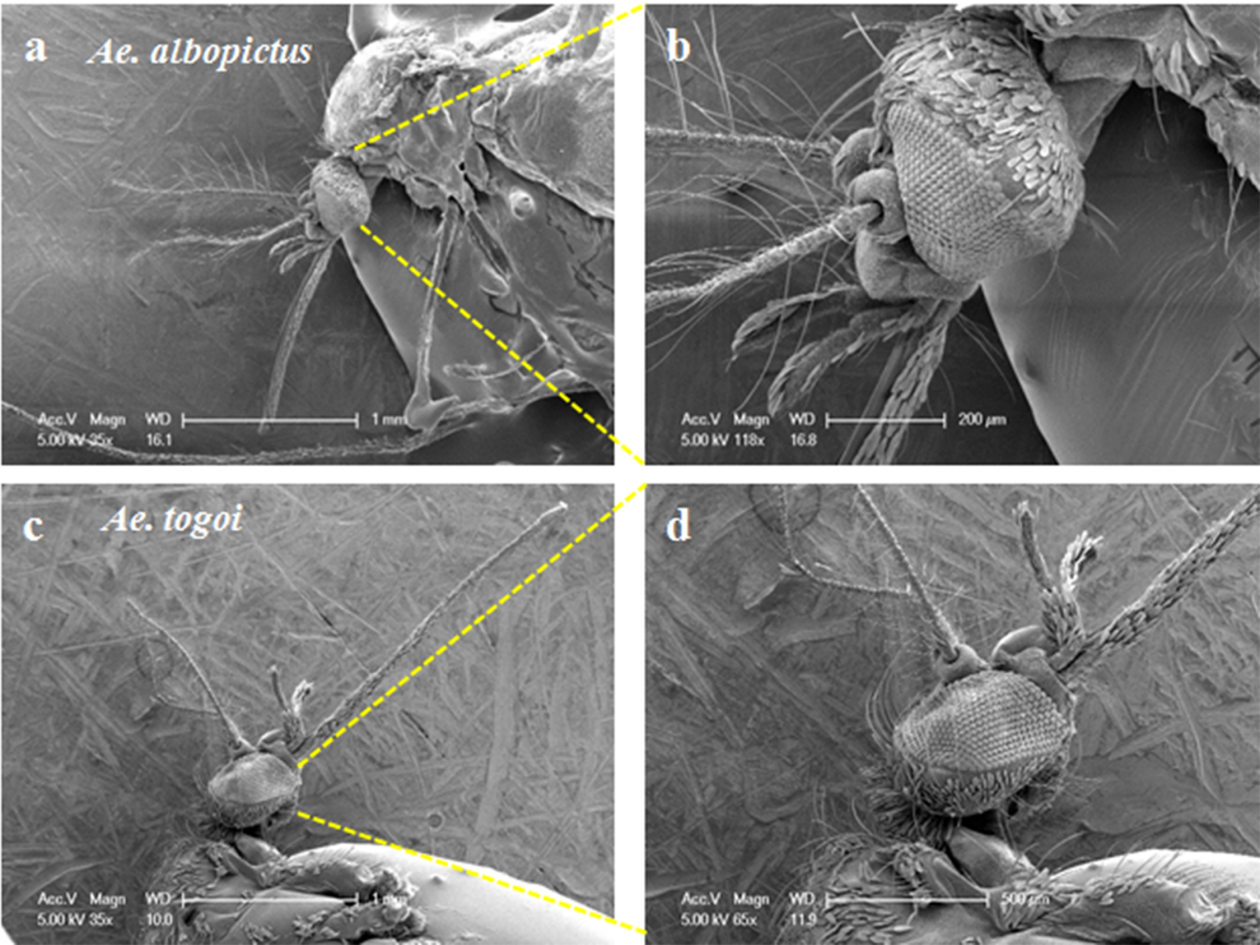
SEM images of the head and proboscis. SEM images of the heads and proboscises of a female *Ae*. *albopictus* (a, b) and *Ae*. *togoi* (c, d) mosquitoes.

A female mosquito sucks blood which contains pathogens through its proboscis by operating two pump organs located in the heads. We examined the morphological characteristics of the proboscises and heads of two female mosquitoes: *Ae*. *albopictus* and *Ae*. *togoi* (Fig 1). The length of the proboscis of *Ae*. *albopictus* is shorter than that of *Ae*. *togoi* and the head size of *Ae*. *albopictus* is smaller than that of *Ae*. *togoi*. The length of the proboscises of two mosquitoes are 1.19 ± 0.13 mm and 2.11 ± 0.05 mm, respectively. The height of their head parts are 0.51 ± 0.04 mm and 0.71 ± 0.05 mm, respectively.

### Comparison of 3D morphological structures of the blood-feeding pump organs

**Fig 2:**
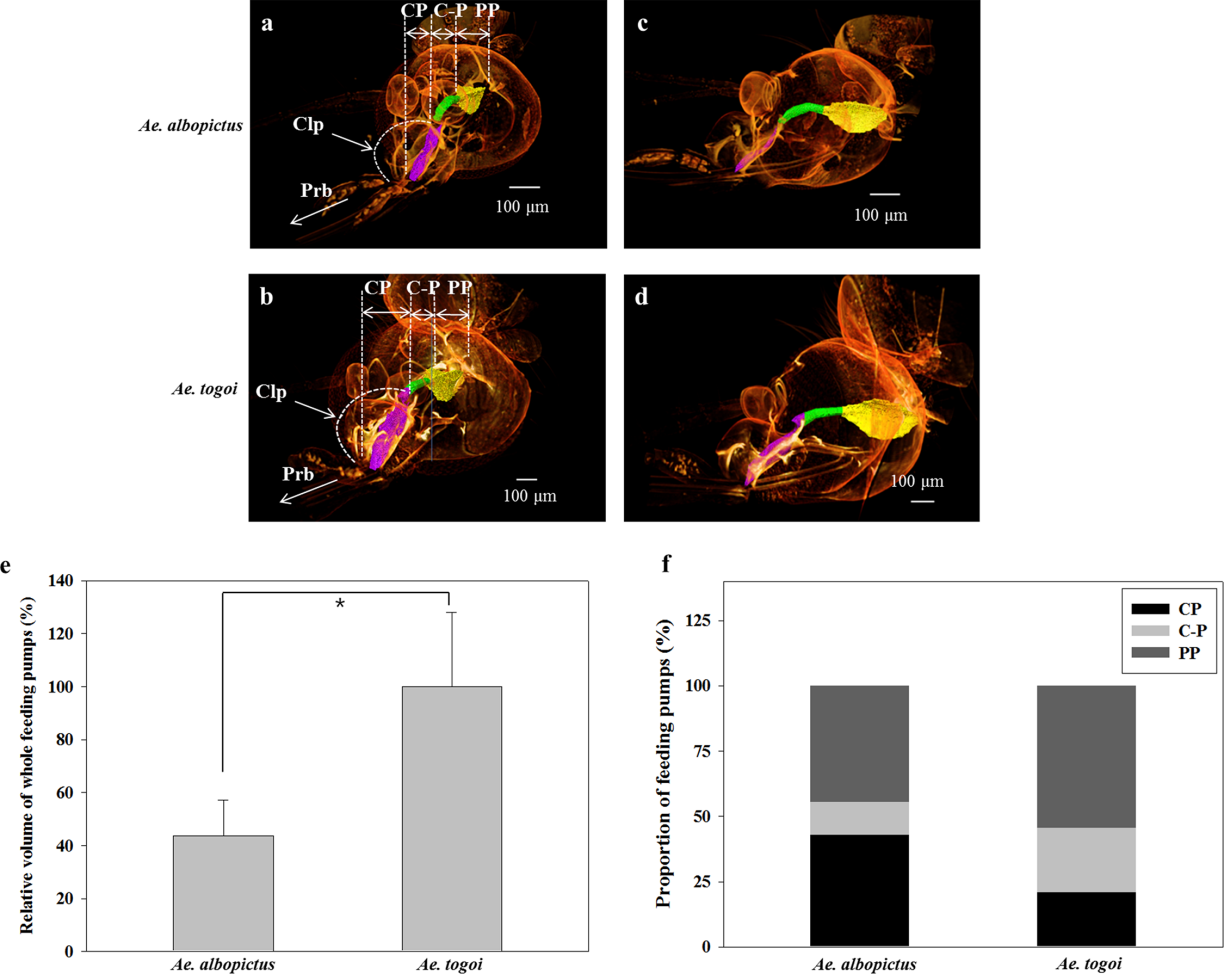
The 3D morphological structures of the heads. The 3D morphological structures of the heads of female *Ae*. *albopictus* (a) and *Ae*. *togoi* (b) mosquitoes reconstructed using SR-μCT. The viewing angle is slightly turned from the coronal view. Sagittal views of *Ae*. *albopictus* (c), and *Ae*. *togoi* (d). (e) Comparison of the total volumes of the two blood-feeding pump organs of *Ae*. *albopictus* and *Ae*. *togoi* mosquitoes. (f) The proportion (%) of three individual feeding pump organs, CP, C-P, and PP to the total volume for both mosquitoes; *p < 0.05.

The 3D morphological structures of the blood-feeding pump organs of two mosquito species under static state were investigated by using synchrotron X-ray computed tomography (Fig 2A to 2D). The blood-feeding pumps of mosquitoes constitute the CP and the PP [13]. The two pump organs are connected by a conduit (C-P) (Fig 2A and 2B). The CP positioned under the curved clypeus is directly linked to the proboscis (Fig 2C and 2D). The PP is located behind the CP and supported by dilator muscles.

The pump organs of *Ae*. *albopictus* are smaller than those of *Ae*. *togoi* at similar developmental stage. The total volume of the two pump organs of *Ae*. *albopictus* and *Ae*. *togoi* is 0.84 ± 0.26 × 10^−3^*mm*^3^ and 1.96 ± 0.54 × 10^−3^*mm*^3^, respectively. The total volume of *Ae*. *albopictus* is about 43%, compared to that of *Ae*. *togoi* (*p* < 0.01) (Fig 2E). The volume of each feeding organ of *Ae*. *albopictus* is also smaller than that of *Ae*. *togoi*. However, the proportions of individual feeding pump organs in *Ae*. *albopictus* are quite different from those in *Ae*. *togoi* (Fig 2F). The proportions of the CP, C-P, and PP volumes are approximately 42.9%, 12.6%, and 44.5%, respectively, in *Ae*. *albopictus*, whereas those in *Ae*. *togoi* are 20.8%, 24.6%, and 54.6%. The volume of CP in *Ae*. *albopictus* took up approximately 40% of the total volume, which is similar to the proportion of PP. The proportion of CP in *Ae*. *albopictus* is relatively high, compared to *Ae*. *togoi*.

### Contraction and expansion of CP during feeding process

**Fig 3:**
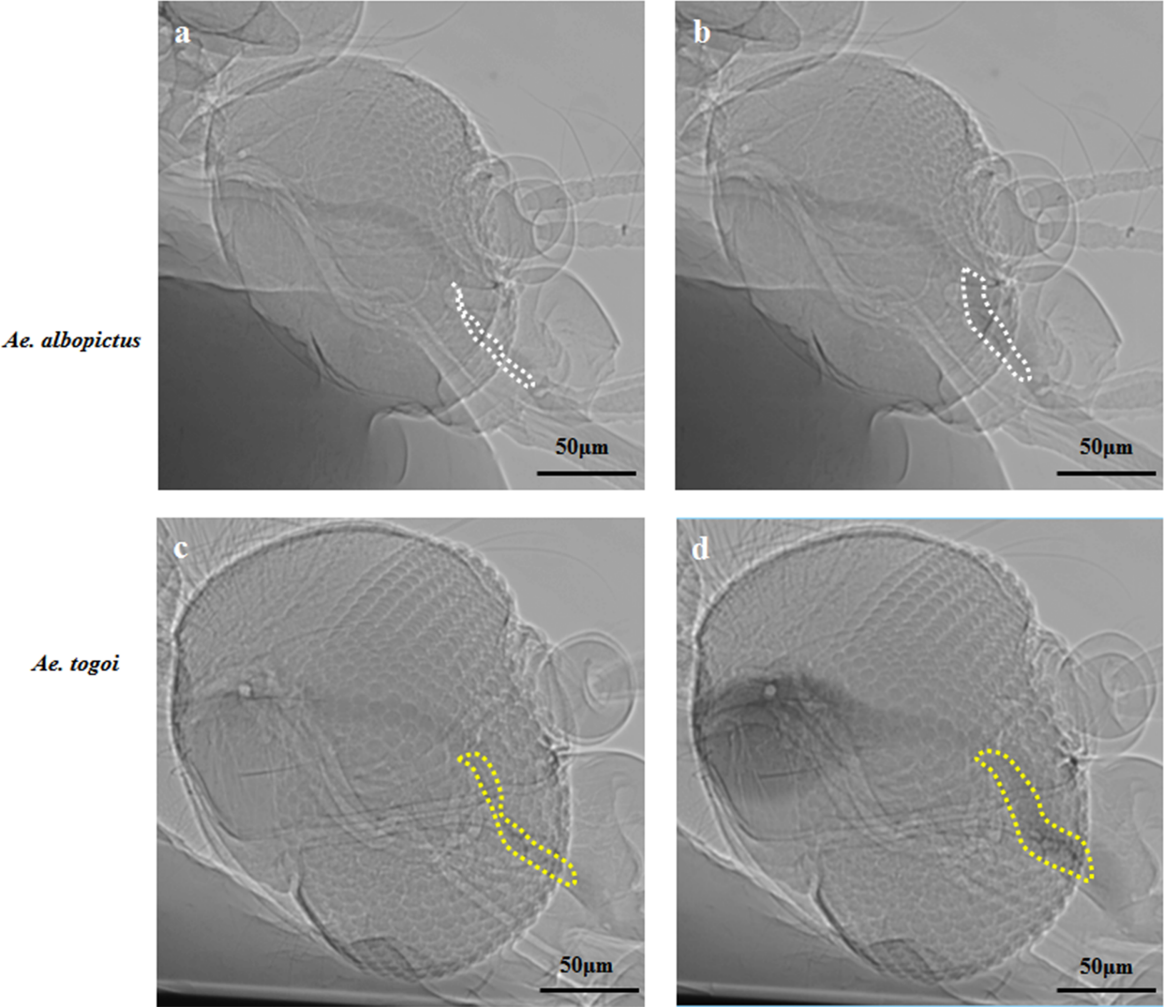
Variations in the CP cross-sectional area. The minimum CP area of *Ae*. *albopictus* (a) and *Ae*. *togoi* (c). The maximum CP area of *Ae*. *albopictus* (b) and *Ae*. *togoi* (d). The cross-sectional CP area was estimated from the X-ray images captured by the synchrotron X-ray micro-imaging technique. A diluted iodine solution was used as the feeding fluid to enhance image contrast.

The consecutive 2D X-ray images of the head parts of two mosquito species show the variations in the cross-sectional area of the CP (Fig 3). The boundaries of the CP are marked with dashed lines to distinguish its maximum and minimum cross-sectional areas. The expansion rate of CP area obtained by dividing the maximum area (A_max_) by the minimum area (A_mim_), are 195.6 ± 8.5% and 145.8 ± 5.4% for *Ae*. *albopictus* and *Ae*. *togoi*, respectively (*p* < 0.01). The volume expansion rates for two different species of mosquito were estimated under assumption of CP as a rotating body. The volumetric expansion rate of CP in *Ae*. *albopictus* is 386.6 ± 31.2% and 228.2 ± 23.5% in *Ae*. *togoi*. The overall expansion rate of CP is much higher in *Ae*. *albopictus* than in *Ae*. *togoi* (*p* < 0.01).

### Feeding characteristics of the two mosquito species

**Fig 4:**
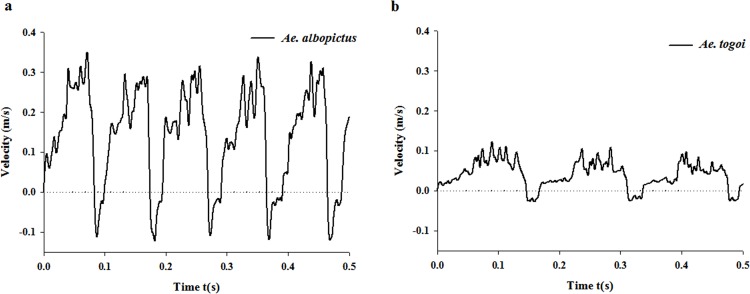
Liquid-feeding flow velocity signals in the food canals. The velocity signals were measured using a micro-PIV technique for *Ae*. *albopictus* (a) and *Ae*. *togoi* (b).

Fig 4 compares the liquid-feeding velocities in the food canals of *Ae*. *albopictus* and *Ae*. *togoi*. Both mosquitoes exhibit periodic liquid suction and ejection characteristics. The start of feeding (t = 0) is determined to be the point when the sign of flow velocity changed from negative (-) to positive (+). The average feeding velocity of *Ae*. *albopictus* is 0.14 ± 0.12 m/s, which was much faster than that of *Ae*. *togoi* (0.04 ± 0.03 m/s) (*p* < 0.01). The pumping frequency of *Ae*. *albopictus* is 10.5 ± 0.2 Hz, which is also faster than in *Ae*. *togoi* (6.1 ± 0.2 Hz) (*p* < 0.01). *Ae*. *albopictus* exhibits fast and frequent liquid sucking, compared to *Ae*. *togoi*.

The liquid flow in the food canal of mosquito during liquid-feeding is analogous to a simple internal flow in a circular tube. The Reynolds number (Re) of the flow inside the food canal is defined as Re = ρu(t)D/μ, where ρ and μ are the density and the dynamic viscosity of the liquid. The averaged velocity u(t) was measured from micro-PIV experiments and the inner diameter (D) of the food canal was obtained from optical microscopic images. The diameters (D) are 22.8 ± 2.5 μm and 27.9 ± 2.3 μm for *Ae*. *albopictus* and *Ae*. *togoi*, respectively (*p* < 0.05). The Re for the liquid flow of *Ae*. *albopictus* and *Ae*. *togoi* is 0.91 ± 0.06 and 0.61 ± 0.09, respectively. The liquid flow in the food canal of both mosquito species is laminar flow because the Re of the flow is less than 2000 which is a critical Reynolds number in a pipe flow. In addition, the Strouhal number (St) of the periodical flow is defined as St = ƒD/u(t), where ƒ is the frequency of vortex shedding. The St is 0.006 ± 0.001 and 0.008 ± 0.001 for the liquid flow of *Ae*. *albopictus* and *Ae*. *togoi*, respectively. The values of Re and the St are sufficiently small to consider the liquid-feeding flow in the food canals of the two mosquitoes in a Hagen-Poiseuille flow.

**Fig 5:**
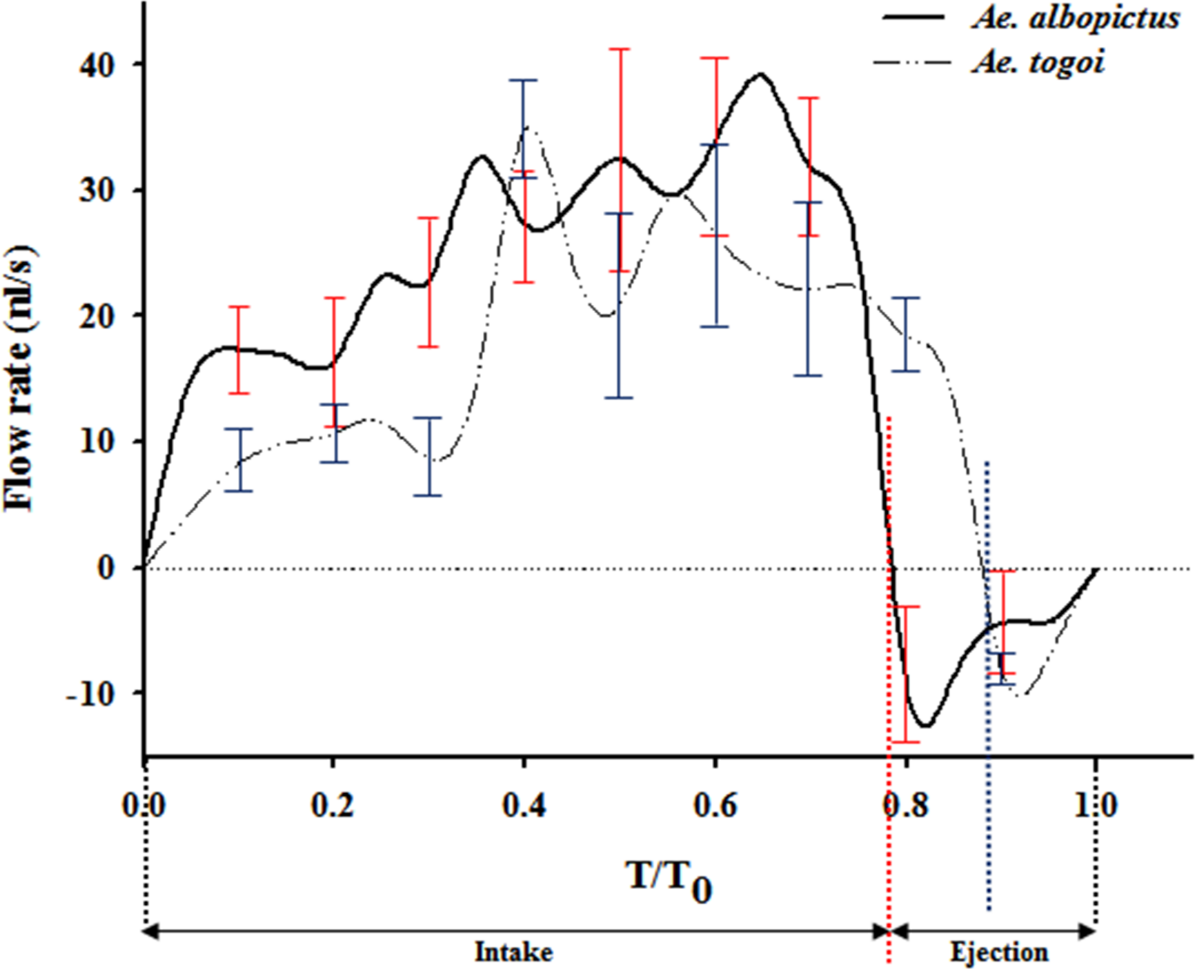
Phasic variations in the flow-rate profiles of the food canal during one pumping cycle. The flow rate was estimated from the velocity field of the liquid-feeding flow, which was measured using a micro-PIV technique assuming Hagen-Poiseuille flow. One liquid-feeding period can be divided into the intake and ejection phases. *T*/*T*_0_ is the dimensionless time normalized by the liquid-pumping period (*T*_0_). The red dotted line indicates the end of the *Ae*. *albopictus* intake phase (*T*/*T*_0_ = 0.767). The blue dotted line indicates the end of the *Ae*. *togoi* intake phase (*T*/*T*_0_ = 0.868). All data are expressed as the mean value ± standard deviation, which is represented by error bars.

To investigate the liquid-feeding characteristics of the two mosquito species, phasic variations of flow rate Q(t) were obtained from the liquid-feeding velocity information in one pumping period (*T*_0_) (Fig 5). Assuming the liquid flow through food canal as the Hagen-Poiseuille flow, the flow rate (Q) can be estimated as follows:

$$Flow\;rate\;Q(t)=\frac{\pi D^{4}\Delta p}{128\mu L}=\frac{\pi D^{2}U_{c}(t)}{8}$$ (1)

where L is the length of the food canal; Δ*p* is the differential pressure generated by the two pumping organs: CP and PP; and *U*_*c*_(t) is the maximum velocity at the center of the food canal. A horizontal axis represents dimensionless time normalized by the pumping period (*T*_0_). The average flow rate of *Ae*. *albopictus* during one feeding cycle (16.8 ± 1.1 nl/s) is relatively higher than that of *Ae*. *togoi* (13.7 ± 1.9 nl/s) (*p* < 0.01). On the other hand, the stroke volume of *Ae*. *Albopictus* (1.64 ± 0.10 nl) during one pumping cycle is much smaller than that of *Ae*. *togoi* (2.24 ± 0.34 nl) (*p* < 0.01).

Interestingly, the liquid-feeding features at the ejection phases of the two mosquito species are quite different. The ejection phase start when 76.7% and 86.8% of the time has elapsed from the start of one cycle for *Ae*. *albopictus* and *Ae*. *togoi*, respectively (*p* < 0.01). However, the average flow rates in the ejection period are quite similar for the two mosquito species; the ejection flow rate of *Ae*. *albopictus* is -6.3 ± 1.0 nl/s and that of *Ae*. *togoi* is -7.2 ± 0.5 nl/s (*p* < 0.01).

We compared the WSS of the two species of mosquito exerted on the wall of food canals during the liquid-feeding process. Assuming that the Hagen-Poiseuille flow holds, WSS (τ_*w*_) was calculated as follows:

$$WSS\;(\tau_{w})=\frac{D∆p}{4l}=\frac{32\mu Q(t)}{\pi D^{3}}$$ (2)

**Fig 6:**
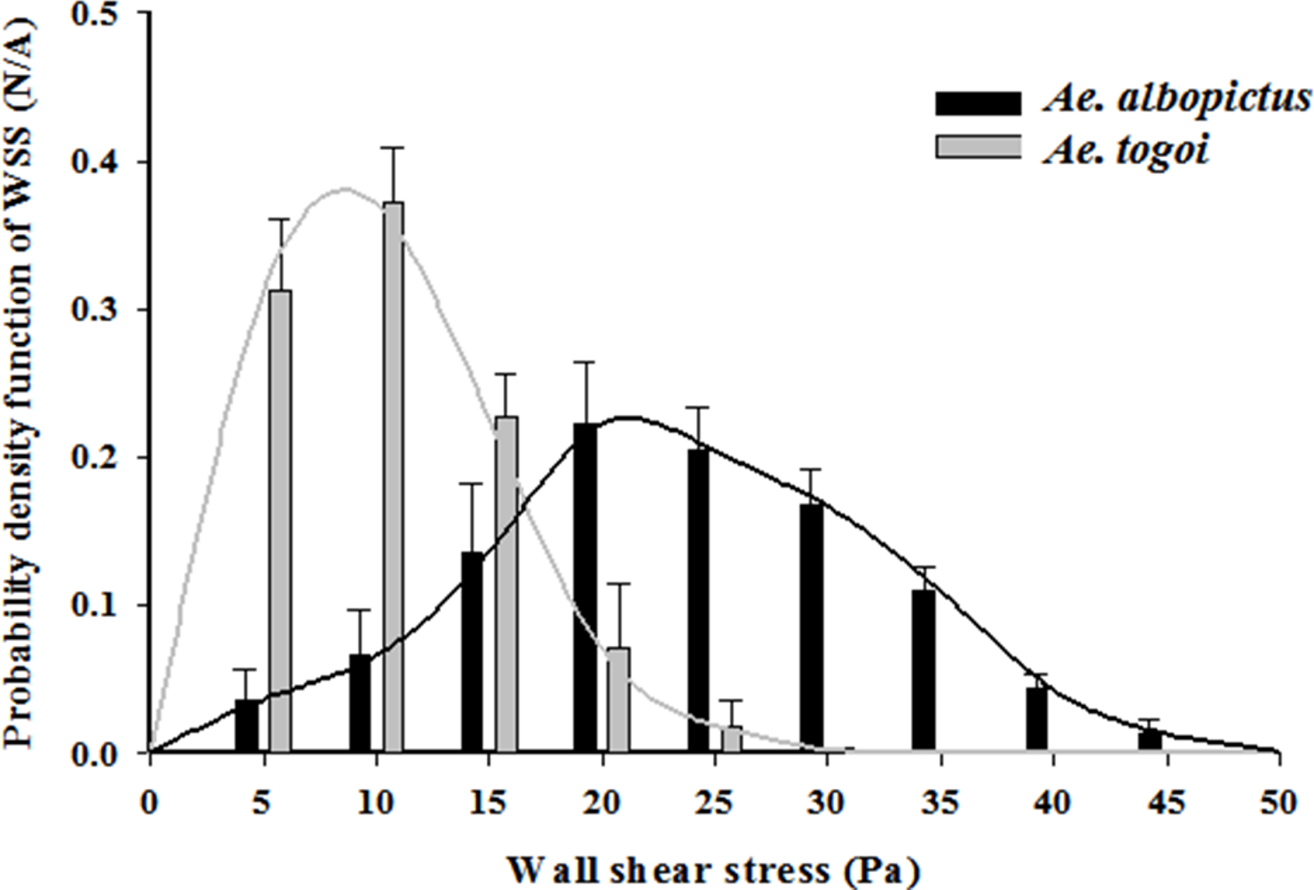
Probability density function (PDF) distribution of WSS during the intake period. The WSS PDF was estimated using the phasic variations of flow rate during the intake period. Standard deviations are represented by error bars.

The WSS increases with increasing flow rate Q(t) and decreasing food canal diameter (D). The probability density function (PDF) distribution of WSS are compared for two different species of mosquito during their intake period (Fig 6). The PDF distribution for *Ae*. *togoi* has the local maximum peak at a relatively low WSS of 8.6 Pa. However, the PDF of WSS for *Ae*. *albopictus* is expressed as Gaussian distribution with a peak at approximately 21.7 Pa. During the liquid intake process, the average WSS of *Ae*. *albopictus* is 21.2 ± 0.4 Pa, which is approximately 2.5 times higher than that of *Ae*. *togoi* (8.2 ± 1.2 Pa) (*p* < 0.01). The WSS of *Ae*. *albopictus* during the entire feeding period (14.9 ± 1.0 Pa) is also much greater than that of *Ae*. *togoi* (6.6 ± 0.9 Pa) (*p* < 0.01).

## Discussion

In this study, we analyzed the liquid-feeding mechanics of the two pumping organs in the head of female mosquitoes: *Ae*. *albopictus* and *Ae*. *togoi*. In particular, we demonstrated that *Ae*. *albopictus*, the specific species of mosquito which is notorious for mediating the transmission of Zika virus, is a fast and gluttonous feeder.

Sant’Anna et al reported that the number of non-ingestive events on the artificial feeder was significantly different from that of observation from live hosts [26]. Thus, it needs to pay attention when we draw conclusions about feeding characteristics from the results acquired by using an artificial meal. In addition, in nematocera diptera, carbohydrate diets are directed to the diverticulum which is attached to the anterior gut rather than to the midgut as it occurs with the blood [27]. However, diet destination can be affected by midgut valve and chemosensory-based control of the crop [28].

*Ae*. *albopictus* rapidly feeds the liquid (1% (w/w) sucrose solution) in spite of a relatively small stroke volume of small sized two-pumps. Kim et al investigated the effect of fluid viscosity on the liquid-feeding of female mosquitoes by measuring the intake flow rate and stroke volume with varying sucrose concentration. They reported that there is no significant difference in the ranges of sucrose concentration between 1% and 30% and 30% and 50% [13]. The total volume of the two pump organs of *Ae*. *albopictus* is found to be approximately half the volume of *Ae*. *togoi*. The stroke volume of *Ae*. *albopictus* is also 73% smaller than that of *Ae*. *togoi*. However, the pumping frequency of *Ae*. *albopictus* is about 1.7 times higher than that of *Ae*. *togoi*. High feeding frequency of *Ae*. *albopictus* means that the species of mosquito can complete the expansion and contraction processes of the pumping organs to fill the pump chamber with liquid in a very short time, compared with *Ae*. *togoi*. This trait may significantly affect the blood feeding and fast pathogen transmission via the blood feeding. *Ae*. *albopictus* is likely to be a more proficient vector because it feeds more rapidly than *Ae*. *togoi*.

The initial instantaneous liquid sucking of *Ae*. *albopictus* is significantly high at the intake phase. The incremental flow rate of *Ae*. *albopictus* during the first 5% of the initial period of one cycle is 15.0 nl/s, which is approximately 300% higher than that of *Ae*. *togoi* (4.5 nl/s). The fast liquid-feeding of *Ae*. *albopictus* at the start of liquid-intake phase may be closely related to the large proportion of the CP to the total pumping system. The volume of CP accounts for a larger proportion in *Ae*. *albopictus*, which means that the variations in volume with the systaltic motion of the CP in *Ae*. *albopictus* is significantly higher than that of *Ae*. *togoi*. The expansion rate of CP area in *Ae*. *albopictus* is approximately 1.3 times larger than that in *Ae*. *togoi*. The faster incremental flow rate of *Ae*. *albopictus* at the start of the intake phase leads to the rapid filling of the CP volume.

*Ae*. *albopictus* has small two-pump chambers, rapidly feeding liquid with small stroke volume, but the accumulated amount of liquid feeding of *Ae*. *albopictus* is 122% larger than that of *Ae*. *togoi* during the same time. The effective liquid-feeding of *Ae*. *albopictus* may be attributed to the similar proportions of its CP and PP. Because both pumps in the two-pump system have the similar size, the mosquito can easily feed and transmit liquid using the same operating frequency [29]. In addition, large expansion rate of CP may contribute to effective pumping performance of *Ae*. *albopictus*. Large displacement of pump chamber can generate the large volume flow rate per stroke [29]. The high WSS of *Ae*. *albopictus* can contribute to vector competence, which represents the infection and transmission abilities of pathogens through blood flow [30]. Adhesive force of pathogen reflects the extraction of elongated tethers by the macrophage membrane. The macrophage membrane immerses the pathogen quickly at the end of initial contact [31]. The shear-induced mechanical drag force in the cell surface cause a high-affinity conformation of the adhesion and decrease the bond off-rate [32]. If the WSS is higher than the adhesive force of the pathogen on the vessel wall, the pathogen can be detached and easily transmitted from hosts to mosquitoes [33]. The extensive WSS might lead to the spread of pathogen by transforming and eventually destroying infected red blood cells [33]. Due to the distinctive WSS difference of *Ae*. *albopictus*, these two *Aedes* mosquitoes potentially have different pathogen transmission efficiencies during sucking.

Mosquito-mediated transmission of Zika virus begins then a *Aedes* mosquito inoculate the virus to the permissive cells of host. Zika virus replication activates an antiviral innate immune response and produces the type Ⅰ interferons in infected cells [34]. The mechanism underlying specific Zika pathogen adhesions will be helpful to understand the adhesive force with WSS of *Ae*. *albopictus*. However, Zika has no vaccines and drugs to treat the infection [35]. Further study of biological factors would be helpful to further understand the pathogen transmission characteristics of *Ae*. *albopictus*.

In this study, the liquid-feeding characteristics and the morphological change of the two-pump system of *Ae*. *albopictus* during liquid-feeding were experimentally investigated and compared with those of *Ae*. *togoi*. This study demonstrates the rapid and gluttonous liquid-feeding characteristics of *Ae*. *albopictus*. Effective liquid feeding of *Ae*. *albopictus* could be explained by structural characteristics and operating characteristics of the pumping organs: similar proportion of CP and PP organs and accelerated liquid intake via fast expanding CP. These results also provide insight into the vector efficiency of *Ae*. *albopictus* in terms of feeding velocity, pumping frequency, intake rate, and WSS.

## Supporting information

S1 File Data set about velocity field information. Liquid-feeding flow velocity signals in the food canals (Fig 4) is based on this data set.
(XLSX)

S2 File Data set about phase-averaged flow rate. Phasic variations in the flow-rate profiles of the food canal during one pumping cycle (Fig 5) is based on this data set.
(XLSX)

S3 File Data set about cumulative wall shear stress (WSS). Probability density function (PDF) distribution of WSS during the intake period (Fig 6) is based on this data set.
(XLSX)
